# Comparison of fear, anxiety and self-efficacy of childbirth among primiparous and multiparous women

**DOI:** 10.1186/s12884-021-04114-8

**Published:** 2021-09-21

**Authors:** Aazam Shakarami, Mojgan Mirghafourvand, Somyieh Abdolalipour, Mohammad Asghari Jafarabadi, Mina Iravani

**Affiliations:** 1 Midwifery Department, Faculty of Nursing and Midwifery, Ahvaz Jundishapur University of Medical Sciences, Ahvaz, Iran; 2 Social Determinants of Health Research Center, Faculty of Nursing and Midwifery, Tabriz University of Medical Sciences, Tabriz, Islamic Republic of Iran; 3 Midwifery Department, Faculty of Nursing and Midwifery, Tabriz University of Medical Sciences, Tabriz, Iran; 4 Department of Statistics and Epidemiology, School of Medicine, Zanjan University of Medical Sciences, Zanjan, Iran; 5 Center for the development of Interdisciplinary Research in Islamic Sciences and Health Sciences, Tabriz University of Medical Sciences, Tabriz, Iran; 6 Midwifery Department, Reproductive Health Promotion Research Center, Faculty of Nursing and Midwifery, Ahvaz Jundishapur University of Medical Sciences, Ahvaz, Iran

**Keywords:** Fear, Anxiety, Self-efficacy, Childbirth, Primiparous women, Multiparous women

## Abstract

**Background:**

The aim of this study was to compare fear of childbirth, state and trait anxiety, and childbirth self-efficacy among primiparous and multiparous women in Ahvaz, southwest of Iran.

**Methods:**

This cross-sectional study was conducted with 200 pregnant women (100 primiparous and 100 multiparous women) who had been admitted to the maternity ward of hospitals affiliated to Ahvaz Jundishapur University of Medical Sciences, Ahvaz, Iran. The instruments used for data collection in this study included a demographic questionnaire, Delivery Fear Scale (DFS), Spielberger's State-Trait Anxiety Inventory (STAI), and Childbirth Self-Efficacy Inventory (CBSEI). The data were analyzed by chi-square test and independent t-test. Also, the univariate general linear model was used by adjusting for the socio-demographic and obstetric characteristics that were considered as possible confounding variables.

**Results:**

The mean score of DFS in primiparous women was significantly higher than that of multiparous women. The mean of the overall score of childbirth self-efficacy of primiparous women was significantly lower than that of multiparous women. The mean score of the outcome expectancies and self-efficacy expectancies was significantly lower in primiparous women compared with multiparous women. There was no statistically significant difference between the two groups in terms of the mean score of STAI. After adjusting for possible confounding variables, the differences between the two groups in terms of fear of childbirth scores, overall childbirth self-efficacy score and self-efficacy expectancies remained significant.

**Conclusion:**

Given the high fear of childbirth and low childbirth self-efficacy in primiparous women compared to the multiparous women, appropriate interventions should be adopted by health care providers in order to reduce fear and improve childbirth self-efficacy in primiparous women.

## Background

Giving birth to a baby is a process accompanied with both joy and pain. In recent decades, labor pains and fears of childbirth have attracted the attention of many researchers who have described childbirth as a very painful phenomenon [1].

According to previous studies, fear of childbirth (FOC) affects about 7.6–8.8% of pregnancies [2, 3] and is more prevalent and more severe in primiparous women compared with multiparous women [4, 5]. Of course, in some studies, the fear of childbirth has been reported to be higher in multiparous women [6, 7]. Parity can affects the content of this fear. Primiparous women tend to be afraid of an unknown pain and their failure to control it. In multiparous women, on the other hand, the fear may arise from previous experiences [8]. Other factors associated with fear of childbirth include complications of previous pregnancies, education level, poor social network, female personality traits such as anxiety, low self-esteem or dissatisfaction with the husband, lack of social and emotional support, and physical and sexual abuse in childhood [7, 9, 10]. The FOC is also associated with adverse maternal outcomes, including poor mental health in the postpartum period and a high frequency of cesarean sections [11, 12].

Until 1990, FOC was studied based on tools used for measuring anxiety such as Manifest Anxiety Scale (MAS) that has three subscales including worry–oversensitivity, fear–concentration, and physiological anxiety. However, psychological domains were later identified for FOC which made anxiety identification instruments less suitable for accurate measurement of this specific fear [13]. Fear of childbirth has been used widely, with no precise definition, but the Wijma Expectancy/Experience Questionnaire Part A (W-DEQ A) and Fear of Birth Scale (FOBS) are two specific instruments that have been used broadly for identification and measurement of FOC. In addition, the main results of study of Wigerta et al. show a deeper understanding of women's definition of FOC, interpreted metaphorically "at a point of no return". It means that women were thinking they could no longer return back to their situation [14]. It can be assumed that women with anxiety are more afraid of childbirth than others [12]. Anxiety activates the sympathetic nervous system, which releases stress hormones, leading to dysfunctional uterine contractions and prolonged labor [15]. Studies have shown that anxiety plays an important role in predicting FOC in women, especially primiparous ones [8]. Çiçek et al. reported that parity can affect childbirth anxiety [16]. However, findings of some other studies have shown that there is no difference between primiparous and multiparous women in terms of state and trait anxiety [17–19]. The review of Rondunget al. reported that FOC is generally positively associated but not overlapped with anxiety (general, state, trait, and sensitivity) [20].

In order to reduce the FOC in women, it is necessary to identify the factors contributing to it. Childbirth self-efficacy may be one of the factors that can play an important role against fear of childbirth. High self-efficacy can reduce this fear and therefore indirectly reduce labor length and the likelihood of the need for epidural analgesia, thus causing better parental outcome [21]. In fact, childbirth self-efficacy can act as a motivating factor for the mother to deal with childbirth, letting the delivery process proceed according to the mother's expectations [22]. Low self-efficacy as well as high levels of FOC, on the other hand, can be considered risk factors contributing to symptoms of post-traumatic stress disorder related to childbirth [23].

Although medical advances in the present century have improved the health of women during pregnancy and childbirth, these advances may have led to the medicalization of almost all deliveries. Thus, the concept of childbirth as a normal psychological phenomenon of life that only occasionally requires intervention has lost its currency [24]. According to the latest systematic reviews, the percentage of cesarean sections in Iran is reported to be 48%, which is much higher than World Health Organization's recommendation (less than 15%) [25]. The results of several studies have also shown that fear, anxiety and self-efficacy during childbirth play an important role in the mother's choice of the type of delivery [23, 26].

Primiparous and multiparous women have been suggested to be different in terms of their childbirth expectations [27]. There may also be differences between primiparous and multiparous women with regard to the perceived natural childbirth process and the actual labor process. Some mothers are frustrated or dissatisfied with the delivery process, while others feel that childbirth process is very surprising and have a positive attitude towards it [28]. Due to the high rate of cesarean section in Iran [25], efforts are being made to investigate, with more details, the reasons for choosing cesarean section by women. To achieve this goal, it is necessary to conduct research that reveals the reasons for women’s reluctance to choose natural based on parity in order to take appropriate interventions. Given the inconsistent results of previous studies and the limited number of studies comparing Iranian primiparous and multiparous women in terms of fear of childbirth, anxiety, and self-efficacy, this study was conducted to compare fear, anxiety and self-efficacy of childbirth among primiparous and multiparous women.

### Hypotheses

- i) The level of fear of childbirth varies between primiparous and multiparous women.
- ii) The level of childbirth anxiety varies between primiparous and multiparous women.
- iii) The level of childbirth self-efficacy varies between primiparous and multiparous women.

## Methods

### Study design and participants

Two hundred pregnant women including 100 primiparous and 100 multiparous women participated in this cross-sectional study. Inclusion criteria were: having a plan for normal delivery according to self-report, being at a gestational age of 36 weeks and more based on the history of obstetrics (last menstrual period and ultrasound during 8 to 12 weeks of pregnancy) and expecting the birth of a healthy and live baby based on obstetrical history and physical examination.

Exclusion criteria were: parity more than 4, experiencing any pregnancy complications requiring medical intervention, receiving anesthesia, having pregnancy risk factors (multiple pregnancy, preeclampsia, etc.) and having an abnormal fetus according to self-report and medical documents.

### Sampling

Convenience sampling was used to select the participants from women referring to one of the university hospitals affiliated to Ahvaz Jundishapur University of Medical Sciences (Razi Hospital), who were admitted for normal delivery and hospitalized in the maternity ward with the diagnosis of labor pains. Pregnant women with spontaneous onset of labor pain during an early stage of active labor (cervical dilation of 3–5 cm) were recruited. After being examined in terms of eligibility criteria, the participants were briefed on the study objectives and methods. In case they were willing to participate in the study and they met the eligibility criteria, written informed consent was obtained from them, and the study questionnaires were completed through interviews with them conducted by the first author. The participants were divided into two groups according to whether or not they had a history of childbirth (primiparous or multiparous). Sampling was continued until the number of samples in both groups was completed.

### Data collection tools

The instruments used to collect the data in this study included a socio-demographic and obstetrics characteristics questionnaire, delivery fear scale (DFS), Spielberger's state and trait anxiety inventory (STAI), and the short form of childbirth self-efficacy inventory (CBSEI).

#### Socio-demographic and obstetrics characteristics questionnaire

This questionnaire was completed using obstetrical medical records and interviews. The questionnaire included questions about a woman's age, her BMI in the first trimester of pregnancy, the woman's and her husband's occupation, her education, economic status, history of infertility, history of abortion, difficult delivery in this pregnancy (dystocia), attendance to childbirth preparation classes, attendance of doula at birth, and having wanted or unwanted pregnancy.

#### Delivery Fear Scale (DFS)

The DFS was designed by Wijma et al. (2002) to assess fear of childbirth during labor [29]. DFS is a 10-item self-assessment questionnaire with scores from 1 (strongly disagree) to 10 (strongly agree). The total score is equal to the sum of the scores obtained from all items, and its range is from 10 to 100. Higher scores indicate more fear. Examples of positively formulated items of DFS include: “I can stand the pain” and “I can manage this”, and those negatively formulated include: “I don’t want to go on any more” and “this is taking forever”. The validity of the Farsi version of this questionnaire has already been assessed and confirmed by our research team (Shakarmi et al.). The internal consistency reliability of this questionnaire was assessed by Cronbach's alpha coefficient which was 0.77 and by split-half coefficient which was 0.83, indicating an acceptable reliability for the questionnaire [30].

#### Spielberger's state-trait anxiety inventory (STAI)

The STAI was developed in 1970 [31]. This questionnaire includes a separate self-assessment scale to measure state and trait anxiety. The state anxiety scale (STAI Form Y-1) consists of twenty sentences that assess a person's feelings at "the moment and the time of response." The trait anxiety scale (STAI Form Y-2) similarly includes twenty items that measure a person's general and normal emotions. Examples of items for state anxiety include: “I feel calm”, “I feel pleasant”, “I feel nervous”, and “I feel jittery”. Examples of items for trait anxiety include: “I am calm, cool and collected", “I am happy”, “I worry too much over something that really doesn't matter”, and “I have disturbing thoughts”. In responding to the state anxiety scale, a number of options are provided for each item, with the participants having to choose the one that best expresses the intensity of their feelings. These options are: 1- (not at all) 2- (somewhat) 3- (moderately so) 4- (so much so). In answering the trait anxiety scale, participants should choose the option that reflects their normal and frequent feelings on a four-level scale as follows: 1- (almost never) 2- (sometimes) 3- (often) 4- (almost always). Each of the STAI test items is assigned a score between 1 and 4 based on the answer provided. STAI has good psychometric properties and is considered as a standard test [31]. Previous studies have shown that all participants except those with personality disorders had higher mean scores on trait anxiety compared with control groups. The mean scores of state anxiety scale have been demonstrated to be higher during stressful situations compared with non-stressful situations [32]. This questionnaire was adapted by the Mortazavi et al. to the Iranian culture and was shown to be a valid instrument for measuring state and trait anxiety in Iranian women [1]. The internal consistency reliability of the questionnaire in this study, based on Cronbach's alpha method, was calculated to be 0.77. Moreover, the Intra-Class Correlation Coefficient (ICC) was 0.77 (95% CI: 0.73 to 0.82).

#### Childbirth self-efficacy inventory

The childbirth self-efficacy inventory was developed by Lowe (1993) [33]. This 62-item self-report instrument (long form) consists of four subscales and two total scales. The subscales are: Outcome Expectancy Active Labor for coping behaviors during active labor (Outcome-AL), items 1–15; Self-Efficacy Expectancy Active Labor for active labor (Efficacy-AL), items 16–30; Expectancy Second Stage for coping behaviors during second stage (Outcome-SS), items 31–46; Self-Efficacy Expectancy Second Stage (Efficacy-SS), items 47–62. The total scores are: the total childbirth outcome expectancy score (total outcome) calculated by summing the Outcome AL and Outcome SS scale scores; and the total self-efficacy expectancy score (total self-efficacy) calculated by summing the Efficacy AL and Efficacy SS scale scores [33]. Outcome expectancy refers to a belief that a particular behavior will produce a particular outcome, and self-efficacy expectancy is the personal belief that one who can successfully perform those behaviors can produce the desired outcome. Scores of total outcome and self-efficacy expectancies may range from 31 to 310 [33]. Because it is too difficult to find out distinct responses between two stages of labor based on repetitive and parallel sets of items in pregnant women, the long form of the questionnaire was modified as a short form [34]. The short form was adapted by Khorsandi et al. to the Iranian culture [35], of course with permission from the author. The short form includes 32 questions in two sections: Sect. 1 (Outcome Expectancies Scale) includes 16 items that measure the expected outcome of childbirth. Section 2 (Self-Efficacy Expectancies Scale) contains 16 questions that measure the expectation of delivery-related self-efficacy. Sample items of this questionnaire include: “I control myself”, “I do not think about the pain”, and “I focus on person helping me in labor”. The items are scored based on a 10-point Likert scale (1 = completely uncertain, to 10 = completely certain), so the scores range from 16 to 160. Higher scores indicate higher outcome expectancies and self-efficacy expectancies. The total self-efficacy score is obtained from the sum of the two scales of outcome expectancies and self-efficacy expectancies [35]. The validity of the questionnaire was confirmed by Khorsandi et al. (2013) for the Iranian community, and its internal consistency reliability based on Cronbach's alpha score was 0.92 for the total scale, 0.88 for outcome expectancies subscale, and 0.88 for self-efficacy expectancies subscale [35]. The internal consistency reliability of the questionnaire in this study was calculated to be 0.86 based on Cronbach's alpha method. Moreover the Intra-Class Correlation Coefficient (ICC) was 0.92 (95% CI: 0.90 to 0.93).

### Sample size

Based on the results of the study of Alehagen et al., [36] and considering 42.80 ± 17.59 (mean ± standard deviation (SD) score of fear of childbirth in primiparous women), 29.51 ± 17.59 (mean ± SD score of fear of childbirth in multiparous women), two sided α = 0.05 and power = 99%, the sample size was calculated to be 66 people using G-power. In this study, the sample size was considered 100 in each group.

### Data analysis

Data collected by questionnaires were entered into IBM SPSS Statistics ver. 24 (IBM Corp, Armonk, USA). The normal distribution of numeric data was assessed and confirmed based on skewness (within ± 1.5) and kurtosis (within ± 2.0). To describe of the socio-demographic and obstetrics characteristics, frequency (percentage) was used for qualitative variables and mean (standard deviation) for quantitative variables. To compare numeric normal and categorical variables between the two groups of primiparous and multiparous women, chi-square and independent t tests were used, respectively. Mean (standard deviation) was used to describe the scores of the Delivery Fear Scale (DFS), State and trait anxiety (STAI), Childbirth Self-Efficacy Inventory (CBSEI) and their subscales. To compare these numeric normal variables between the two groups, independent t-test was used, and the univariate general linear model was used by adjusting for the socio-demographic and obstetrics characteristics that were considered as possible confounding variables (*P* < 0.2). Parity was entered as independent variable. Age, BMI, economic status, education, intention of pregnancy, and history of abortion were entered as confounder variables, whereas fear of childbirth, total childbirth self-efficacy and its domains (outcome expectancies and self-efficacy expectancies), state anxiety and trait anxiety were entered as dependent variables. Significance level was set at *α* = *0.05*.

## Results

The maternal characteristics stratified by parity are shown in Table 1. Twelve multiparous women (12%) had a history of previous dystocia.

**Table 1:** Characteristics of the study participants (*n* = 200)

Variable	Primiparous (*n*=100)	Multipara (*n*=100)	*P*-Value
**Age** (years) *Mean (SD)*	24.6 (5.3)	29.8 (5.9)	<0.001^†^
**BMI** at first trimester (kg/m^2^) *Mean (SD)*	26.7 (4)	28.1 (4.3)	0.014^†^
**Education**			0.017^¥^
Primary school	13 (13)	19 (19)	
Secondary school	33 (33)	38 (38)	
High school Diploma	34 (34)	36 (36)	
Bachelor	17 (17)	7 (7)	
Master and higher	3 (3)	0 (0)	
**Occupation***n (%)*			0.099*
Housewife	88 (88)	96 (96)	
Employee	5 (5)	1 (1)	
Worker	7 (7)	3 (3)	
**Spouse’s occupation***n (%)*			0.549^§^
Employee	13 (13)	9 (9)	
Worker	24 (24)	25 (25)	
Self-employment	42 (42)	50 (50)	
Others	21 (21)	16 (16)	
**Economic status***n (%)*			<0.001^¥^
Weak	44 (44)	33 (33)	
Moderate	50 (50)	33 (33)	
Good	6 (6)	34 (34)	
**Infertility***n (%)*			0.721*
No	97 (97)	95 (95)	
Yes	3 (3)	5 (5)	
**Wanted pregnancy***n (%)*			<0.001*
No	0 (0)	19 (19)	
Yes	100 (100)	81 (81)	
**Abortion***n (%)*			<0.001^§^
0	96 (96)	79 (79)	
1	4 (4)	21 (21)	
**Participation in childbirth preparation classes***n (%)*			0.004*
No	68 (68)	85 (85)	
Yes	32 (32)	15 (15)	
**Attendance of doula during delivery***n (%)*			0.311*
No	99 (99)	97 (97)	
Yes	1 (1)	3 (3)	

^†^ Independent Samples T Test; ^*^Fisher's Exact Test; ^§^Chi-square test, ^¥^ Chi-square for trend

The comparison of the scores of primiparous and multiparous women obtained from delivery fear scale, total childbirth self-efficacy and its subscales, and state and trait anxiety inventory is displayed in Table 2. The primiparous women’s mean score from DFS was significantly higher than that of multiparous women. Multiparous women had higher scores in terms of total childbirth self-efficacy, self-efficacy expectancies, and outcome expectancies compared with primiparous women. There were no significant differences between the two groups in terms of the mean scores of state and trait anxiety.

**Table 2:** Comparison of the scores of delivery fear scale, total childbirth self-efficacy and its subscales, state and trait anxiety in primiparous and multipara women

**Variable**	**Primiparous** (*n* = 100)	**Multipara** (*n* = 100)	Comparison between groups^*^	
	Mean (SD^†^)	Mean (SD^†^)	MD (95% CI)^‡^	*P*-Value
**Delivery Fear Scale**	69.3 (8.5)	42 (8.8)	27.3 (24.9 to 29.7)	< 0.001
**Total self-efficacy**	207.3 (43.9)	222.4 (39.0)	-15.1 (-26.7 to -3.5)	0.011
**Outcome expectancies**	103.5 (22.0)	110.3 (20.5)	-6.8 (-12.7 to -0.9)	0.024
**Self-efficacy expectancies**	103.8 (22.0)	112.2 (19.3)	-8.3 (-14.1 to -2.5)	0.005
**State anxiety**	40.9 (5.0)	41.7 (5.7)	-0.9 (-2.3 to 0.6)	0.264
**Trait anxiety**	40.2 (5.3)	41.2 (5.7)	-1.0 (-2.5 to 0.5)	0.207

^*^ Independent t-test; ^†^ Standard Deviation; ^‡^ Mean Difference (95% Confidence Interval)

The comparison of the above scales is displayed in Table 3 based on univariate general linear model after adjustment for variables including age, BMI, economic status, level of education, intention of pregnancy, and the history of abortion, which were significantly different between the two groups. The mean score of fear of childbirth was significantly higher in primiparous women than in multiparous women. The mean scores of total self-efficacy and self-efficacy expectancies were significantly lower in primiparous women compared with multiparous women. There was no statistically significant difference between the two groups in terms of the mean score of outcome expectancies and the score of state and trait anxiety.

**Table 3:** Comparison of the scores of delivery fear scale, total childbirth self-efficacy and its subscales, state and trait anxiety in primiparous and multipara women based on the general linear model

Variable	B* (95% Confidence Interval)	***P***-Value
**Delivery Fear Scale**		
Primiparous	26.9 (23.8 to 30)	< 0.001
Multipara (Reference)	0	
**Total childbirth self-efficacy**		
Primiparous	-16 (-31.4 to -0.83)	0.039
Multipara (Reference)	0	
**Outcome expectancies**		
Primiparous	-7.8 (-14.9 to 0.6)	0.069
Multipara (Reference)	0	
**Self-efficacy expectancies**		
Primiparous	-9.3 (-16.8 to -1.8)	0.016
Multipara (Reference)	0	
**State anxiety**		
Primiparous	-1.8 (-3.8 to 0.11)	0.379
Multipara (Reference)	0	
**Trait anxiety**		
Primiparous	-1.9 (-3.2 to 0.8)	0.240
Multipara (Reference)	0	

^*^Values have been adjusted for age, BMI, education of mother, economic status, wanted pregnancy, abortion history and participation in childbirth preparation classes

## Discussion

The hypotheses of the present study were that the levels of fear, anxiety and self-efficacy of childbirth vary between primiparous and multiparous women. According to the results of the present study, the mean score of fear of childbirth in primiparous women was significantly higher than that in multiparous women. In terms of mean total self-efficacy, outcome expectancies, and self-efficacy expectancies, primiparous women had significantly lower scores compared with multiparous women. There was no significant difference between the two groups in terms of the mean score of state and trait anxiety. After adjustment for age, BMI, economic status, mother's education level, intention of pregnancy, and history of abortion, there was a statistically significant difference between the two groups in terms of the mean score of fear of childbirth, total self-efficacy score, and self-efficacy expectancies score.

In the present study, the mean score of fear of childbirth was higher in primiparous women compared with multiparous women. Consistent with the results of the present study, in some previous studies, fear of childbirth has been found to be more severe in primiparous women than in multiparous women [4, 5]. Of course, there are studies that have different results which are inconsistent with those of the present study. Nillson et al., for instance, despite reporting a higher rate of fear of childbirth among primiparous women than multiparous women, found that this difference was not statistically significant [2]. Moreover, according to some other studies such as Raisanen et al. [17] and Khwepeya et al. [37], a higher percentage of multiparous women as opposed to their primiparous counterparts reported severe fears of childbirth. Fears of multiparous women compared with primiparous women may be the result of a previous traumatic childbirth and indicate that they suffer from post-traumatic stress disorder (PTSD) in the postpartum period [8]. In their study comparing the experiences and expectations of childbirth in primiparous and multiparous women, Pirdel et al. reported that fear of childbirth in both groups was one of the most important factors giving rise to negative experiences and expectations about childbirth [27]. Early identification of women at risk of childbirth fear is of clinical significance for improving women's health care during pregnancy and postpartum period. Therefore, clinical attention to fear of childbirth along with comprehensive assessments and mental health care is essential [37].

In the present study, the mean score of childbirth self-efficacy in primiparous women was significantly lower than that of multiparous women. There are a number of studies that are in line with the results of our study in terms of childbirth self-efficacy scores after adjusting for possible confounding variables. Lowe et al., for example, found no significant difference between the two groups in terms of outcome expectancies score. However, in terms of self-efficacy expectancies and total self-efficacy, multiparous women scored significantly higher than primiparous women [33]. Moreover, in some other studies, the self-efficacy expectancies score in multiparous women was significantly higher than that of primiparous women [21, 38]. Other studies have examined the self-efficacy of women undergoing natural childbirth only among primiparous women [24, 25, 39, 40]. In all of these studies, primiparous women with high self-efficacy scores had lower fears of childbirth.

According to the theory of self-efficacy, an individual may believe that certain behaviors can facilitate their adaptation to an unfavorable situation, but they may doubt their ability to perform those behaviors [23]. Since beliefs about self-efficacy are essential for cognitive regulation of motivation, women with low self-efficacy may have limited ability to motivate themselves to adapt to the experience of childbirth. If a woman does not believe in her ability to perform the tasks or make efforts necessary to prepare her for labor, she will be unlikely to be even motivated to try this. Of course, the socio-cultural climate in which a woman's sense of self-efficacy for childbirth is nurtured needs to be taken into account [28].

In the present study, there was no difference between primiparous and multiparous women in terms of state and trait anxiety, which is consistent with the results of numerous other studies [17–19]. However, Çiçek et al. reported that parity can affect childbirth anxiety [16]. Differences in the level of anxiety in pregnant women may be related to factors other than parity. While some anxious women may prefer to be hospitalized quickly for their reassurance, some may delay hospitalization if the hospital setting is an anxious environment [41].

Given that the fear of childbirth can affect the choice of delivery method, measuring the level of fear and anxiety and determining the level of self-confidence and self-efficacy of women in pregnancy can help members of the health care team to identify those women who request cesarean section out of fear or anxiety [42]. During labor, professional support by the midwife and other maternity staff can enhance a woman's sense of self-efficacy and help her become more adapted to the situation and avoid triggering negative emotions [43].

The findings of this study can provide important insights into women's experiences of childbirth and the emotional aspects of childbirth. Therefore, knowing these experiences can be used to evaluate and improve reproductive health policies and services. For example, this could be achieved by the implementation of some appropriate interventions for each woman such as execution of birth plan according to women's preferences, which seems to have been neglected in most settings.

Despite using standard and valid tools in this study, which is one of its strengths, there are a number of limitations to take into account. First of all, the cross-sectional design of the study and the convenience sampling method may have affected the results obtained. Failure to study the factors affecting the fear and anxiety of pregnant women such as socio-economic status, psychological status, marital satisfaction, social support, etc. can be regarded as another limitation. Also, using non-specific tool to measure anxiety was another limitation. Therefore, longitudinal studies investigating participants from the very beginning of pregnancy in terms of the factors affecting the mode of delivery are recommended.

## Conclusion

Given the high fear of childbirth and low childbirth self-efficacy in primiparous women compared to the multiparous women, appropriate interventions such as implementation of birth plan according to women's preferences should be taken into account by health care providers in order to decrease this fear and improve childbirth self-efficacy in these women.

## Data Availability

The datasets used and/or analyzed during the current study are available from the corresponding author upon reasonable request.

## Abbreviations

DFS: Delivery Fear Scale
STAI: Spielberger's State-Trait Anxiety Inventory
CBSEI: Childbirth Self-Efficacy Inventory
FOC: Fear of childbirth
